# Transcatheter arterial embolization using n-butyl cyanoacrylate metacryloxysulfolane for traumatic or iatrogenic non-visceral hemorrhage

**DOI:** 10.3389/fradi.2026.1878048

**Published:** 2026-06-25

**Authors:** Paolo Federico Garducci, Bernardo Proner, Timo Alexander Auer, Florian Nima Fleckenstein, Francesco Giurazza, Pietro Roccatagliata, Claudio Carruba, Romaric Loffroy, Federico Collettini

**Affiliations:** 1 Department of Radiology, IRCCS Azienda Ospedaliero-Universitaria Di Bologna, Bologna, Italy; 2 Department of Diagnostic and Interventional Radiology, Charité Universitätsmedizin Berlin, Berlin, Germany; 3 Department of Vascular and Interventional Radiology, Cardarelli Hospital, Naples, Italy; 4 Department of Vascular and Interventional Radiology, François-Mitterrand University Hospital, Dijon, France

**Keywords:** hemorrhage, iatrogenic bleeding, n-butyl cyanoacrylate, transarterial embolization, trauma

## Abstract

**Purpose:**

To evaluate the safety and effectiveness of transcatheter arterial embolization (TAE) using n-butyl cyanoacrylate metacryloxysulfolane (NBCA-MS) as a first-line embolic agent for the management of traumatic and iatrogenic non-visceral arterial hemorrhages in an emergency setting.

**Materials and methods:**

This retrospective study included all consecutive patients who underwent selective TAE with NBCA-MS for non-visceral hemorrhage of traumatic or iatrogenic origin between June 2021 and June 2025. All patients were assessed with contrast-enhanced multidetector computed tomography (CT) demonstrating active arterial bleeding or pseudoaneurysm prior to embolization. TAE was performed via a femoral approach using selective microcatheterization, followed by embolization with NBCA-MS mixed with ethiodized oil at variable dilutions. Technical success was defined as complete angiographic occlusion of the bleeding vessels. Clinical success was defined as sustained hemostasis without need for repeat endovascular or surgical intervention /within 30 days. Procedure-related complications were recorded according to CIRSE guidelines.

**Results:**

Fifty-four patients (37 males; median age 60.5 years) underwent 54 TAE procedures. Traumatic hemorrhage accounted for 83.3% of cases, and iatrogenic hemorrhage for 16.7%. A single artery was embolized in 72.2% of procedures. Technical success was achieved in all cases (100%). Clinical success was observed in 46 patients (85.2%). Six patients required repeat embolization due to hemorrhage from vessels within the same vascular territory that had not been bleeding during the initial procedure and were therefore not embolized at that time. No procedure-related deaths, major complications, or off-target embolizations were observed.

**Conclusion:**

NBCA-MS-based TAE is a safe, effective, and versatile treatment for traumatic and iatrogenic non-visceral arterial hemorrhage. Its high technical success, favorable clinical outcomes, and excellent safety profile support its use as a reliable first-line embolic agent in urgent clinical scenarios.

## 1 Introduction

Traumatic and iatrogenic non-visceral hemorrhages constitute a broad group of bleeding events that occur outside the solid abdominal organs. They are defined as bleeding originating from soft tissues, the body wall, musculofascial compartments, and branch vessels of the thoracic, abdominal, pelvic, or peripheral arterial trees—such as intercostal, epigastric, lumbar, gluteal, and muscular branches. Within this spectrum, traumatic non-visceral hemorrhage arises from blunt or penetrating injury, whereas iatrogenic non-visceral hemorrhage results directly from diagnostic or therapeutic procedures (e.g., biopsy or drain placement). In the trauma setting, uncontrolled hemorrhage remains a leading cause of preventable mortality, and non-visceral sources can be brisk, occult, and physiologically consequential, particularly in older patients, those receiving antithrombotic therapy, or those with trauma-induced coagulopathy (1–4). Iatrogenic hemorrhage, meanwhile, reflects the growing volume and complexity of diagnostic and therapeutic procedures. Although many iatrogenic bleeds are self-limited, a clinically meaningful subset progress to hemodynamic instability, transfusion dependence, or compartment syndrome, demanding timely definitive hemostasis. Management principles emphasize early recognition, physiological stabilization, and rapid control of the bleeding source (5). While surgery remains indispensable in selected scenarios, transarterial embolization (TAE) has emerged as a cornerstone modality for many non-visceral bleeding sources owing to its speed, precision, and organ-sparing profile (6). TAE achieves hemostasis by super-selectively catheterising the culprit artery (or arterial territory) and deploying an embolic agent to occlude the site of bleeding (7). A variety of embolics are available, each with distinct mechanical and hemodynamic properties. Coils and vascular plugs provide proximal mechanical occlusion; particles and microspheres offer distal embolization; and liquid agents create rapid, durable casts capable of penetrating complex or fragile vascular beds (7–9). Among liquids, n-butyl cyanoacrylate metacryloxysulfolane (NBCA-MS) has particular theoretical and practical advantages in the acute bleeding context: it polymerizes rapidly on contact with ionic solutions, provides permanent hemostasis independent of the patient's coagulation status, can be titrated (via dilution with radiopaque ethiodized oil) to control depth of penetration, and is widely available (9, 10). These characteristics are attractive for traumatic and iatrogenic non-visceral hemorrhage, where friable vessels, vasospasm, or coagulopathy can limit the effectiveness of mechanical or particulate agents. Despite the growing use of TAE across trauma systems and peri-procedural care pathways, data specifically addressing NBCA-MS as the first-line embolic for traumatic and iatrogenic non-visceral hemorrhage remain limited. To address this knowledge gap, the present study evaluates selective TAE using NBCA-MS as a first-line embolic agent for the management of non-visceral hemorrhage of traumatic and iatrogenic origin.

## 2 Materials and methods

The imaging studies and clinical records of all consecutive patients who underwent TAE using NBCA-MS as the first-line embolic agent for the treatment of non-visceral hemorrhages of traumatic or iatrogenic origin between June 2021 and June 2025 were collected and retrospectively reviewed. The study was reviewed and approved by the local institutional review board.

### 2.1 Preinterventional evaluation and indication for transarterial embolization

All patients had a clinical diagnosis of active bleeding and underwent contrast-enhanced multidetector computed tomography (MDCT) to confirm the presence and location of the hemorrhage. Patients were referred for angiography and subsequent embolization if MDCT demonstrated active contrast extravasation consistent with arterial bleeding or arterial pseudoaneurysm. The indication for TAE was established by an interdisciplinary team comprising anesthesiologists, surgeons, interventional radiologists, and members of the emergency medical team. The decision was based on a combination of factors, including the patient's hemodynamic status, ongoing transfusion requirements, comorbidities, and the anatomical feasibility of embolization.

### 2.2 Interventional technique

TAE procedures were performed either under general anesthesia or under local anesthesia with anesthesiology standby, depending on the overall clinical condition and stability of the patient. Awake and responsive patients were always informed of the potential risks of the intervention before the procedure, despite its emergency nature, including the possibility of reintervention and non-target embolization. All patients received appropriate hemodynamic support throughout the intervention, including intravenous fluids, blood product transfusions, and vasopressors when indicated. Vascular access was obtained via a common femoral artery approach using a 4-F or 5-F introducer sheath. An initial nonselective diagnostic arteriogram of the aorta was performed using a pigtail catheter to localize the bleeding site and assess for additional vascular injuries. Once the hemorrhage was identified, a diagnostic catheter (e.g., C2 Cobra, Simmons-1, or Mikaelsson) was positioned in the relevant feeding artery. Through this catheter, a microcatheter (Cantata™, Cook Medical; Progreat™, Terumo) was advanced over a microwire (e.g., Fathom™, Boston Scientific) to achieve a selective approach to the bleeding vessel. Digital subtraction angiography was performed through the microcatheter to identify the bleeding site and exclude collateral vessels that could lead to non-target embolization, such as the anterior spinal artery or radiculomedullary branches during intercostal artery embolization. Before embolization, the microcatheter was flushed with 5% dextrose solution to prevent premature polymerization of the glue within the catheter lumen. Embolization was then performed under continuous fluoroscopic monitoring using NBCA-MS (Glubran 2, GEM Srl) mixed with ethiodized oil (Lipiodol Ultra Fluid; Guerbet) in ratios ranging from 1:1 to 1:5, tailored according to the vascular territory, flow dynamics, and degree of microcatheter selectivity in order to optimize glue penetration and minimize proximal reflux. After delivery of the NBCA-MS mixture, the microcatheter was promptly withdrawn to minimize the risk of catheter entrapment. A final, non-selective control angiogram was obtained in all cases to confirm complete occlusion of the target vessel and cessation of contrast extravasation.

### 2.3 Study endpoints and definitions

Technical success was defined as complete angiographic occlusion of all target vessels responsible for the hemorrhage at the end of the procedure. Clinical success was defined as effective control of bleeding without the need for any further endovascular or surgical intervention within 30 days after embolization. Clinical success was assessed by the absence of a renewed decline in hemoglobin levels, no new episodes of overt bleeding, and no imaging or clinical evidence suggestive of ongoing or recurrent hemorrhage following the TAE procedure.

Clinical follow-up was based on a combination of clinical evaluation, serial hemoglobin measurements, hemodynamic monitoring, and review of medical records during the 30-day follow-up period. Routine imaging follow-up was not systematically performed in all patients due to the retrospective nature of the study. However, additional contrast-enhanced CT and/or repeat angiography were obtained whenever recurrent bleeding was clinically suspected based on hemodynamic deterioration, decreasing hemoglobin levels, or new signs of overt hemorrhage.

Any recurrent bleeding arising from the original bleeding site within 30 days after the initial embolization and necessitating repeat intervention (either repeat TAE, surgery, or another hemostatic procedure) was classified as a clinical failure. Procedure-related complications were recorded and graded according to the classification system of the European Society of Cardiovascular and Interventional Radiology (CIRSE) Standards of Practice Committee (11).

### 2.4 Statistical analysis

Statistical analysis was performed using IBM SPSS Statistics for Macintosh, version 27.0 (IBM Corp, Armonk, NY, USA). Data were described as frequencies for categorical variables, mean values with standard deviations for normally distributed continuous data, and as medians [interquartile range, IQR (Q1, Q3) for non-normally distributed continuous data.

## 3 Results

The study population consisted of 54 patients (37 males; with a median age of 60.5 years) who underwent 54 TAE procedures. The demographic data of the patients and the procedural details are summarized in Table 1.

**Table 1:** Baseline patient characteristics and procedural parameters.

Parameter	Value
Median age; range (years)	60.5; 26–94
Gender (male/female)	37/17
Traumatic hemorrhage	45
Iatrogenic hemorrhage	9
Number of embolized vessels	
One artery	39
Two arteries	13
Three arteries	2
Embolized arteries	
Intercostal artery	32
Inferior epigastric artery	8
Deep and superficial circumflex iliac artery	7
Lumbar artery	7
Internal thoracic artery	6
Lateral thoracic artery	3
Medial and lateral circumflex femoral artery	2
Dorsal scapular artery	1
Iliolumbar artery	1
NBCA-MS dilution with ethiodized oil	
1:1	0
1:2	19
1:3	30
1:4	4
1:5	1
Median [IQR] fluoroscopy time (minutes)	13.51 [7.10–21.24]
Median [IQR] dose–area product (Gy·cm^2^)	53 [26.6–187.26]

**Figure 1:**
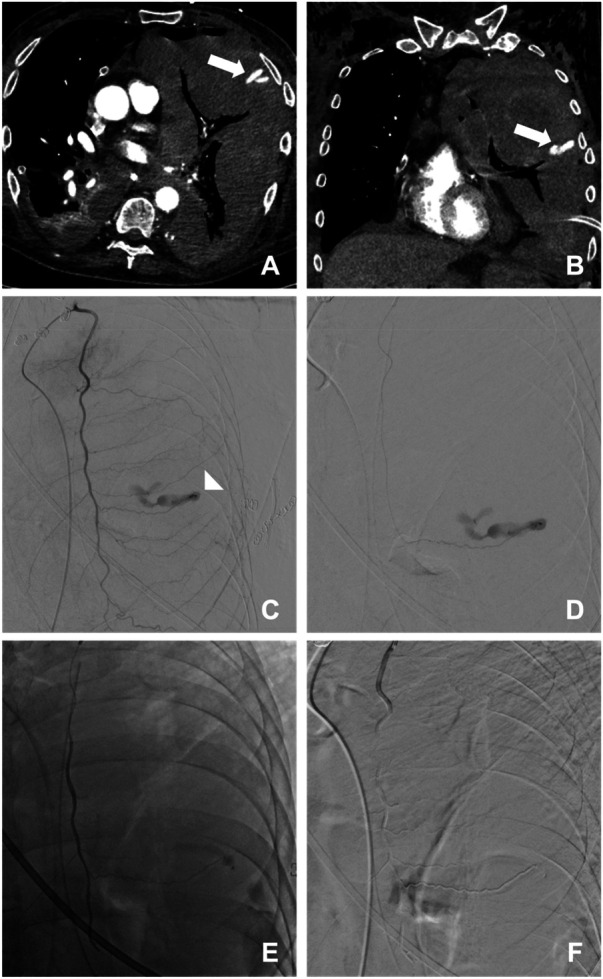
A 68-year-old man with sequelae of polytrauma. **(A,B)** Axial and coronal contrast-enhanced CT images demonstrate active contrast extravasation from the left internal thoracic artery secondary to a rib fracture (bold arrows). **(C)** Selective angiography confirms active bleeding arising from an anterior intercostal branch (arrowhead). **(D)** Superselective catheterization of the bleeding vessel is performed. **(E)** Embolization is achieved using n-butyl cyanoacrylate mixed with ethiodized oil in a 1:2 ratio. **(F)** Final angiographic control demonstrates complete and successful embolization of the internal thoracic artery with no evidence of residual bleeding.

The predominant indication for TAE was traumatic hemorrhage, which was present in 45 patients (83.3%) (Figure 1). The remaining 9 patients (16.7%) underwent TAE for iatrogenic bleeding occurring after diagnostic or therapeutic procedures, including pleural drain placement, biopsy procedures, or the insertion of an extracorporeal membrane oxygenation (ECMO) device. In most procedures, embolization was limited to a single arterial branch (39 procedures, 72.2%). In the remaining 15 patients (27.8%), embolization of multiple arteries was required to achieve hemostasis: two arteries were embolized in 13 patients (24.1%), and three arteries in 2 patients (3.7%).

The analysis of embolized targets revealed a heterogeneous distribution of bleeding sources. The intercostal arteries emerged as the most frequent site of intervention, being treated in 32 patients (59.3%). Inferior epigastric arteries represented the second most common source (8 cases, 14.8%), whereas the lumbar arteries and deep or superficial circumflex iliac arteries were involved with same frequency, in 7 patients each (13%). The internal thoracic artery accounted for 6 cases (11.1%). Finally, in a further 7 patients (13%), embolization was directed towards others less common arterial territories, which included the lateral thoracic artery, the medial and later circumflex femoral artery, the dorsal scapular artery and the iliolumbar artery. With respect to the dilution of NBCA-MS with ethiodized oil, the 1:3 ratio represented the most frequently adopted mixture, used in 30 procedures (55.6%). This was followed by the 1:2 ratio in 19 cases (35.2%), the 1:4 ratio in 4 cases (7.4%), and the 1:5 ratio in one single case (1.9%). Notably, no procedures were performed using a 1:1 dilution. The median dose–area product (DAP) expressed in Gy·cm² and providing an estimate of the overall patient radiation exposure, was 53 Gy·cm² (IQR 26.6–187.26). The median fluoroscopy time was 13.5 min (IQR 7.10–21.24).

### 3.1 Technical success

A completion angiogram obtained immediately after each embolization confirmed complete embolization of all angiographically identified target vessels supplying the hemorrhage in every patient. Accordingly, the technical success rate of the procedure was 100%. Throughout all interventions, there were no episodes of inadvertent adhesion or “gluing” of the microcatheter to the vessel wall or embolic material, and in every case the microcatheter could be retrieved in its entirety without complication.

### 3.2 Clinical success

**Figure 2:**
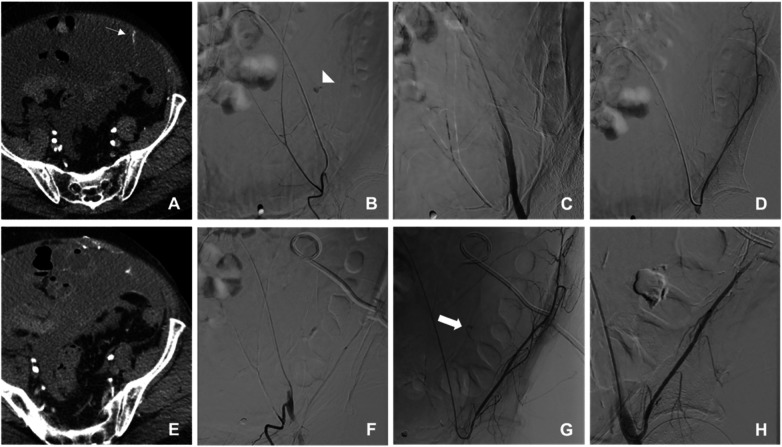
A 57-year-old patient with alcoholic cirrhosis complicated by acute-on-chronic liver failure, with clinical suspicion of iatrogenic hemorrhage following ascites paracentesis. **(A)** Axial contrast-enhanced CT image demonstrates active contrast extravasation arising from the left inferior epigastric artery (thin arrow). **(B)** Selective angiography confirms active bleeding from a branch of the left inferior epigastric artery (arrowhead), **(C)** which was successfully treated by embolization using n-butyl cyanoacrylate mixed with ethiodized oil in a 1:3 ratio. **(D)** Selective angiography of the ipsilateral deep circumflex iliac artery shows no evidence of active bleeding. **(E)** One day after embolization, the patient again developed clinical signs of hemorrhage and repeat contrast-enhanced CT demonstrated new active contrast extravasation in the same anatomical region. **(F)** Repeat angiography confirmed persistent occlusion of the left inferior epigastric artery following prior embolization, **(G)** but revealed new active contrast extravasation from a branch of the ipsilateral deep circumflex iliac artery (bold arrow). **(H)** This newly identified bleeding source was successfully embolized using n-butyl cyanoacrylate mixed with ethiodized oil in a 1:3 ratio.

Clinical success of embolization defined as effective control of bleeding without the need for any further endovascular or surgical intervention within 30 days after embolization was achieved in 46 of 54 patients (85.2%). None of these 46 patients required any additional invasive procedures to achieve or maintain hemostasis throughout the follow-up period. Of the remaining eight patients, two patients (3.7%) died the day after embolization despite effective embolization—one from pericardial tamponade and the other from massive endobronchial bleeding. The remaining six patients (11%) experienced new hemorrhages originating from vessels within the same vascular territory that had not been bleeding during the initial procedure and were therefore not embolized at that time (Figure 2). All these cases were managed successfully with repeat sessions of NBCA-MS-based TAE.

### 3.3 Procedure-related complications

There were no deaths attributable to the procedure, and no major complications occurred as a consequence of the embolization. More specifically, there were no instances of non-target embolization, and no spillover or unintended migration of glue was detected either during the interventions or on follow-up imaging studies.

## 4 Discussion

The present study investigates the role of NBCA-MS-based TAE as a first-line interventional therapy for the management of non-visceral hemorrhage of traumatic and iatrogenic origin. By selecting a clearly defined patient population and applying a consistent NBCA-MS-centered embolization protocol, this work provides focused, practice-oriented evidence on the performance of this technique in a real-world emergency setting. Within this context, our results demonstrate that NBCA-MS-based TAE is both safe and effective for the treatment of non-visceral hemorrhage. Technical success was achieved in all treated patients, with completion angiography confirming complete occlusion of all target vessels. Clinical success at 30 days—defined as durable hemostasis without the need for repeat endovascular or surgical intervention—was obtained in 46 of 54 patients (85.2%). None of the 46 patients required additional invasive procedures to achieve or maintain hemostasis during the follow-up period. Among the remaining eight patients, two (3.7%) died on the day following embolization despite technically successful treatment, one due to pericardial tamponade and the other due to massive endobronchial hemorrhage. The remaining six patients (11%) developed new bleeding episodes arising from vessels within the same vascular territory that had not exhibited active hemorrhage during the initial procedure and were therefore not embolized. Among the six patients who required repeat embolization, 2 of 6 procedures had been performed by less experienced operators/trainees under supervision (one and three years of experience), whereas the remaining 4 of 6 were performed by senior interventional radiologists with 6 or more years of experience in embolization procedures. Importantly, in all six cases, recurrent hemorrhage did not result from technical failure, incomplete embolization, or recanalization of the treated vessel, but rather from newly evident bleeding arising from adjacent vessels within the same vascular territory that were angiographically occult during the initial procedure.

Importantly, there were no procedure-related deaths, no major complications, and no radiological or clinical evidence of non-target embolization or glue migration. (Figure 2).

Cyanoacrylate is an established liquid embolic agent that has gained increasing use for transarterial embolization in non-visceral bleedings, including musculoskeletal, soft-tissue, and post-traumatic hemorrhage (12). Its mechanism of action is based on rapid polymerization upon contact with ionic solutions such as blood, resulting in an immediate and durable vascular occlusion (13). This property makes cyanoacrylate particularly attractive in hemodynamically unstable patients or in those with underlying coagulopathy, where the efficacy of particulate or coil embolization may be reduced. The role of cyanoacrylate as a liquid embolic agent in acute hemorrhage is well established in guidelines and expert reviews, which emphasize its usefulness when superselective catheterization is difficult, long vessel segments must be occluded, or the patient is coagulopathic (1, 2, 8, 10, 14). In the setting of non-visceral bleeding, cyanoacrylate is especially useful for the embolization of small-caliber, tortuous, or distally located arterial branches that are difficult to access or to occlude effectively with coils. Compared with other embolic materials, cyanoacrylate offers several advantages: it is independent of the patient's coagulation status, provides immediate occlusion, and has a low risk of recanalization because of its permanent polymerized cast (15). In addition, relatively small volumes of cyanoacrylate are often sufficient to achieve hemostasis, and the procedure time can be shortened, which is beneficial in emergency scenarios (9). N-butyl cyanoacrylate–metacryloxysulfolane (NBCA-MS) is a cyanoacrylate adhesive in which NBCA is copolymerized with metacryloxysulfolane to retain the rapid, permanent cast formation characteristic of NBCA while improving handling properties and biocompatibility relative to conventional NBCA formulations (9). A key practical advantage is the reduced exothermicity during polymerization: whereas classic NBCA may polymerize with comparatively high heat release, incorporation of the MS comonomer lowers the peak polymerization temperature to approximately 45 °C (often contrasted with ∼90 °C reported for conventional NBCA in the endovascular literature). This attenuated thermal profile has been associated with reduced perivascular inflammation and histotoxicity and may contribute to lower procedure-related pain and local tissue irritation in selected vascular territories. In addition, NBCA-MS is frequently described as less prone to catheter “grab” at the microcatheter tip. Although it remains an adhesive liquid embolic, the copolymer is reported to form a more pliable and stable polymer network with less abrupt polymerization behavior than some standard NBCA products, translating into improved injectability and a more controllable penetration–reflux balance, consistent with the perception of reduced “stickiness” (9). For intramuscular active hemorrhages, Yoo et al. reported 18 patients treated with NBCA-based TAE, achieving 100% technical success, 83% clinical success, and no serious procedure-related complications (16). More recently, Albuquerque et al. described 11 patients with life-threatening abdominal wall hemorrhage undergoing NBCA-based TAE, again with 100% technical success, high clinical success (63%), and no embolic-agent–related adverse events (17). Similarly, Moretti et al. evaluated 47 polytrauma patients with active abdominopelvic bleeding treated exclusively with NBCA, reporting 100% technical and clinical success, absence of rebleeding, and no embolization-related complications (6, 18). Outside the trunk, several series support NBCA use for peripheral traumatic and iatrogenic vascular lesions. Oh et al. described three cases of hemorrhage from branches of the superficial femoral artery after blunt trauma treated with superselective NBCA embolization, with immediate bleeding control and preserved limb perfusion (19). In the renal vasculature, Kim et al. reported a 14-patient series with varied renal vascular lesions treated by NBCA embolization, demonstrating 100% technical success and 85.7% clinical success, with minimal complications limited to one case of global renal atrophy and one microcatheter tip fracture without clinical sequelae (20). Morsi et al. presented a case series of 13 patients with traumatic face and neck vessel injuries, predominantly from gunshot wounds, in whom NBCA embolization achieved immediate hemostasis in all cases; there was no rebleeding, one ischemic complication, and low mortality despite the severity of trauma (21).

Our results align with previously published evidence and further support the efficacy and safety of TAE using NBCA-MS. We observed a technical success rate of 100% and a clinical success rate exceeding 85%, demonstrating that embolization was successfully accomplished in all cases and resulted in meaningful clinical improvement for most patients (46 of 54). Among the eight patients without clinical success, two died the day following the TAE procedure despite technically effective embolization, while the remaining six developed recurrent hemorrhage from vessels within the same vascular territory that were not actively bleeding at the time of the initial intervention and therefore had not been embolized.

It should be emphasized that none of the repeat embolization procedures were attributable to recanalization or technical failure of previously embolized vessels. In all six cases, follow-up imaging and repeat angiography confirmed persistent occlusion of the initially treated arteries, while the recurrent hemorrhage originated from newly identified bleeding vessels within the same vascular territory that had not demonstrated active extravasation during the initial procedure.

Importantly, these outcomes were achieved in urgent and emergency clinical contexts, where procedures are inherently more challenging due to limited preparation time, hemodynamic instability, and the necessity for rapid, high-stakes decision-making. In addition, embolizations were performed by multiple operators with different levels of experience and across different vascular territories, suggesting that—when appropriate technique and standardized protocols are applied—the procedure is reproducible and not excessively dependent on individual operator expertise. Equally important, we observed no procedure-related adverse events, including no off-target embolization, which is frequently cited as a concern with NBCA due to its rapid polymerization and the theoretical risk of non-target migration. Our results therefore provide pragmatic evidence that, with careful handling and adequate supervision, NBCA-MS-based TAE can be implemented safely even in academic environments, where trainees and less-experienced interventionalists may participate as part of structured teams. Several limitations should be acknowledged. The study's retrospective, single-center design and the relatively small sample size may limit generalizability. Furthermore, the analyzed cohort was heterogeneous with respect to bleeding etiology and target vessels, which may introduce confounding and complicate interpretation.

Moreover, as this study was designed as a retrospective, non-comparative analysis of glue embolization, the inclusion of patients in whom glue was selected at the operator's discretion may have introduced selection bias and may limit the generalizability of the findings to procedures performed with other embolic agents.

In addition, the inclusion criteria required CT evidence of active arterial bleeding or pseudoaneurysm prior to angiography, which may have introduced a degree of selection bias. Consequently, the present findings may not be fully generalizable to patients with intermittent hemorrhage or angiographically occult bleeding patterns not detected on preprocedural imaging.

Finally, the absence of a comparative arm (other embolic materials) restricts direct benchmarking and limits conclusions about relative performance across different approaches.

## 5 Conclusions

In summary, our data support NBCA-MS embolization as a safe, effective, and versatile option for the management of non-visceral arterial bleeding in both traumatic and iatrogenic contexts. Despite the study limitations, the combination of uniform technical success and favorable clinical outcomes highlights NBCA-MS as a reliable embolic agent in time-sensitive scenarios. Larger, ideally prospective and comparative studies are warranted to confirm these findings and to better characterize the long-term safety profile and clinical durability of cyanoacrylate-based embolization.

## Data Availability

The raw data supporting the conclusions of this article will be made available by the authors, without undue reservation.
